# A Localized Surface Plasmon Resonance Sensor Using Double-Metal-Complex Nanostructures and a Review of Recent Approaches

**DOI:** 10.3390/s18010098

**Published:** 2017-12-31

**Authors:** Heesang Ahn, Hyerin Song, Jong-ryul Choi, Kyujung Kim

**Affiliations:** 1 Department of Cogno-Mechatronics Engineering, Pusan National University, Busan 46241, Korea; ahn3890@pusan.ac.kr (H.A.); rin@pusan.ac.kr (H.S.); 2 Medical Device Development Center, Daegu-Gyeongbuk Medical Innovation Foundation (DGMIF), Daegu 41061, Korea; jongryul32@dgmif.re.kr; 3 Department of Optics and Mechatronics Engineering, Pusan National University, Busan 46241, Korea

**Keywords:** sensors, surface plasmon resonance (SPR), localized surface plasmon resonance (LSPR), long-range surface plasmon resonance (LRSPR), double-metal-complex

## Abstract

From active developments and applications of various devices to acquire outside and inside information and to operate based on feedback from that information, the sensor market is growing rapidly. In accordance to this trend, the surface plasmon resonance (SPR) sensor, an optical sensor, has been actively developed for high-sensitivity real-time detection. In this study, the fundamentals of SPR sensors and recent approaches for enhancing sensing performance are reported. In the section on the fundamentals of SPR sensors, a brief description of surface plasmon phenomena, SPR, SPR-based sensing applications, and several configuration types of SPR sensors are introduced. In addition, advanced nanotechnology- and nanofabrication-based techniques for improving the sensing performance of SPR sensors are proposed: (1) localized SPR (LSPR) using nanostructures or nanoparticles; (2) long-range SPR (LRSPR); and (3) double-metal-layer SPR sensors for additional performance improvements. Consequently, a high-sensitivity, high-biocompatibility SPR sensor method is suggested. Moreover, we briefly describe issues (miniaturization and communication technology integration) for future SPR sensors.

## 1. Introduction

A sensor is defined as any device that converts one or more types of information, such as pressure, velocity, acceleration, temperature, frequency, and biological signals, into readable quantitative signals [1,2,3]. From active developments and applications of various devices to acquire inside and outside information and to operate based on feedback from that information, the sensor market is rapidly growing. The global sensor market is predicted to grow from 795.5 billion in 2014 to 1161 billion dollars in 2019, and the average annual growth rate is expected to be approximately 7.9% [4]. For example, biosensors, which are analytical devices for detecting specific biological components, are actively used in medical, environmental, industrial, military, food and research areas [5,6,7,8,9,10]. A car or an aircraft may be equipped with many sensors, such as an oxygen sensor, an air temperature sensor, an air mass sensor and a position sensor, and these sensors are utilized for various purposes, ranging from driver protection to fuel efficiency improvements [11,12,13].

As an optical sensor, the surface plasmon resonance (SPR) sensor has been developed for high-sensitivity real-time detection [14,15,16,17,18,19]. The SPR sensor is exploited to detect biological/chemical interactions in the SPR sensing area by measuring characteristic changes in the medium, i.e., the refractive index. Due to their sensitivity and selectivity with the capability of label-free detection, SPR sensors have been used for the detection and analysis of various biomolecules, such as proteins, nucleic acids, and viruses [10,11,12,13,14,15,16,17,18,19,20,21,22]. Moreover, SPR sensors are applicable to analyze variations or interactions in live cells in real-time [23,24,25,26,27,28]. Live cell analysis has attracted much attention in drug discovery and development, with the aim of understanding the biological functions of cells. Furthermore, SPR sensors have been used to acquire specifically targeted information from specimens, for instance, the chemical composition or status of hazardous material retention [29,30].

First, this article provides a report on the fundamentals of SPR sensors and recent approaches for enhancing sensing performance. In the section on the fundamentals of SPR sensors, a brief description of surface plasmon resonance (SPR) phenomena, SPR-based sensing applications, and several configuration types of SPR sensors are introduced. In addition, advanced nanotechnology- and nanofabrication-based techniques for improving the sensing performance of SPR sensors are proposed: (1) localized SPR (LSPR) using nanostructures or nanoparticles; (2) long-range SPR (LRSPR); and (3) double-metal-layer SPR sensors for additional performance improvements. Finally, we suggest a performance-enhanced LSPR sensor method integrated with a double-metal-complex nanostructure to enhance the sensitivity and biocompatibility in a practical way without biofunctionalization. In addition, a brief prospect of how SPR sensors can be integrated with technologies during the current phase of the 4th industrial revolution is suggested in the concluding remarks.

## 2. SPR Sensors

### 2.1. Fundamentals of SPR and SPR-Based Sensors

A plasmon is defined as a quasiparticle generated by collectively vibrating electrons in a metal [31,32,33,34]. A plasmon is commonly located on the surface of a metal. Thus, a plasmon distributed on a metal surface is called a surface plasmon. When light at a specific wavelength is shone on a metallic layer with a specific thickness, a collaborative interaction between the surface plasmon and a photon occurs. This surface plasmon condition is called SPR, and a quasiparticle derived from the interaction of the surface plasmon and the photon is defined as a surface plasmon polariton (SPP).

Derived from Maxwell’s equations and the Drude model, a model is used to determine the behavior of electrons in a metal based on a kinetic theory [35,36]. The incidence and characteristics of SPR are sensitively related to various factors such as photon wavelength, incident illumination angle, metal type, metallic layer thickness, and the optical properties of dielectric materials above the metallic layer. From this property, SPR can be applied as a sensor to detect the variance of optical properties in dielectric specimens on a metallic surface. SPR sensors monitor refractive index changes of a medium by measuring shifts in the resonance angle (angular interrogation) [37,38,39,40] or resonance wavelength (wavelength interrogation) [41,42,43,44] in the output optical signal. The sensitivity of SPR sensors is determined using the following parameters: sharpness of resonance, resonant spectral shift, and figure of merit (FOM).

### 2.2. Classification of SPR Sensors

#### 2.2.1. Prism-Based Method

**Figure 1:**
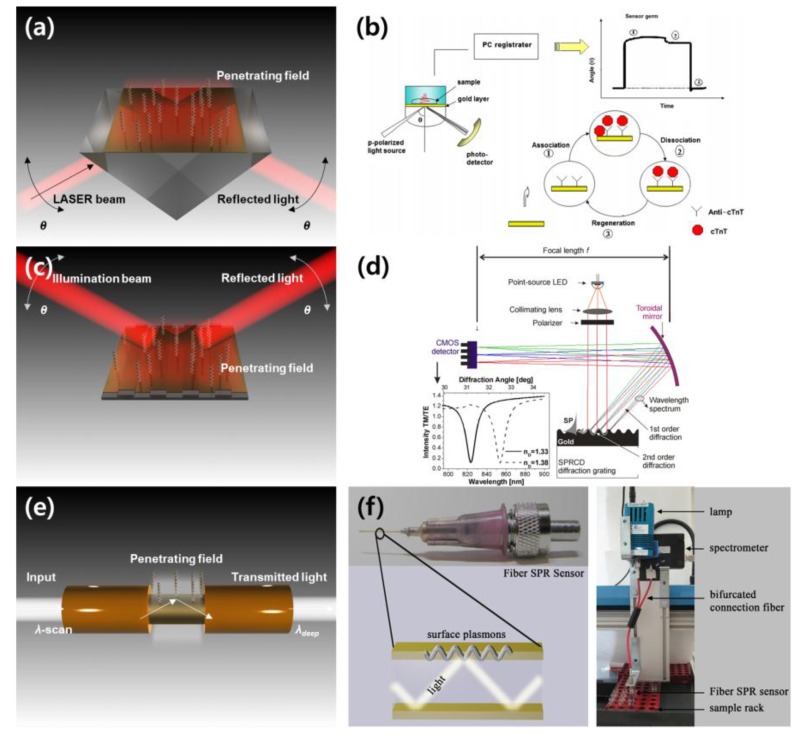
(**a**) Schematic of a prism-based SPR sensor with the Kretschmann configuration; (**b**) schematic illustration of the principle of a prism-based SPR immunoassay to determine concentrations of cardiac troponin T (reproduced from [46] with permission from Elsevier); (**c**) Schematic of a diffraction grating-based SPR sensor; (**d**) a miniaturized, diffraction grating-based SPR sensor for highly sensitive refractive index estimation of the specimen above the grating (reproduced from [47] with permission from Elsevier); (**e**) schematic of a fiber-based SPR sensor; (**f**) a fiber-based SPR sensor that is compatible with a commercial syringe needle to detect DNA hybridization processes and a prototype of an automatic multi-sample DNA hybridization instrument with the integration of computer-controlled sensor positioning stages (reproduced from [48] with permission from Elsevier).

According to the SPR generation processes on a metal layer described in the previous section, light should illuminate and provide an excitation of the surface plasmon with a finely tuned angle and wavelength. The use of a prism to create total internal reflection (TIR) coupling is one appropriate method for securing SPR conditions. Practical, well-estimated prism-based SPR sensors are classified into Otto and Kretschmann configurations. In the Otto configuration of prism-based SPR sensors, there is a certain distance between the prism and the metal layer on which the light is employed. Between the prism and the metal layer, there is a dielectric layer with a smaller refractive index. In the Kretschmann configuration, the metallic layer is in direct contact with the prism, as shown in Figure 1a. Because there is a difference in the distance between the metal layer and the prism surface on which TIR occurs, Kretschmann configuration-based SPR sensors normally have a higher efficiency than Otto configuration-based sensors [45].

From a previous study on the sensing performance of Kretschmann configuration-based SPR sensors for samples with a changing refractive index [49], the sensitivities of SPR sensors that detect by measuring the resonance angle (*S_θ,p_*) and spectral wavelength (*S_λ,p_*) shifts were determined using the following equations:

(1) $$S_{\theta ,p}=\frac{Re\left(\varepsilon_{metal}\right)\sqrt{-Im\left(\varepsilon_{metal}\right)}}{\left\{Re\left(\varepsilon_{metal}\right)+{\left(n_{analyte}\right)}^{2}\right\}\sqrt{Re\left(\varepsilon_{metal}\right)\left\{{\left(n_{analyte}\right)}^{2}-{\left(n_{prism}\right)}^{2}\right\}-{\left(n_{p6rism}\right)}^{2}{\left(n_{analyte}\right)}^{2}}}$$

(2) $$S_{\lambda ,p}=\frac{{\left\{Re\left(\varepsilon_{metal}\right)\right\}}^{2}}{\left\{\frac{{\left(n_{analyte}\right)}^{3}}{2}\right\}\left|\frac{dRe\left(\varepsilon_{metal}\right)}{d\lambda }\right|+\left\{Re\left(\varepsilon_{metal}\right)+{\left(n_{analyte}\right)}^{2}\right\}Re\left(\varepsilon_{metal}\right)\frac{dn_{prism}}{d\lambda }\frac{n_{analyte}}{n_{prism}}}$$

where *ε_metal_* is the metal permittivity, *n_analyte_* is the refractive index of the specimen on the SPR sensor, and *n_prism_* is the refractive index of the prism in the Kretschmann configuration.

SPR sensors that use prisms have been actively implemented for sensitive detection of various biological, chemical and environmental changes. Dutra et al. suggested an SPR sensor using the Kretschmann configuration for the cardiac troponin T, a biomarker that indicates cardiac diseases such as myocardial infarctions, as shown in Figure 1b [46]. In this study, a linear response and an exclusive reproducibility in sensing the cardiac troponin T at concentrations from 0.05 to 4.5 ng/mL were achieved. Prabhakar et al. investigated the highly sensitive *Mycobacterium tuberculosis* using a prism-based SPR sensor [50]. A peptide nucleic acid (PNA) probe that can detect *Mycobacterium tuberculosis* was immobilized on the surface of a gold layer deposited above a BK7 glass substrate, and the SPR sensor using PNA probes provided efficient *Mycobacterium tuberculosis* detection. Horiuchi et al. integrated capillary tubes to a prism-type SPR sensing instrument for an on-site immunoassay (antibody-antigen attachment) analysis [51]. Successful detection of specific antibody-antigen binding excluding non-specific reactions was confirmed in a preliminary study, and the feasibility of application for biomedical or environmental high-sensitivity sensors was suggested. de Juan-Franco et al. developed a novel preparation and binding method using a protein A-gold binding domain for improving SPR immunosensors and investigated the performance of human growth hormone sensing in prism-based SPR sensing [52]. As an example of an environmental gas sensor using different wavelengths, Herminjard et al. introduced an SPR carbon dioxide (CO_2_) sensor using a mid-infrared (IR) wavelength (*λ* = 4.4 μm) and a CaF_2_ prism-based Kretschmann configuration [53]. In comparison with a visible SPR CO_2_ sensor, the sensitivity of the mid-IR SPR CO_2_ sensor was improved 5-fold.

#### 2.2.2. Diffraction Grating-Based Method

As another method, several research groups have investigated SPR sensors using a diffraction grating instead of a prism. A diffraction grating is an optical device that diffracts light in several directions, which can be determined by the amplitude, period, and optical characteristics of the grating material. In addition, the refractive index of a superstrate on the diffraction grating affects the characteristics of the directions of diffracted light. From this property, a diffraction grating with a metal deposition can be integrated in SPR sensors, as illustrated in Figure 1c. Homola et al. estimated the sensitivity of diffraction grating-based SPR sensors, which perform detection by measuring the resonance angle (*S_θ,g_*) and spectral wavelength (*S_λ,g_*) shifts:

(3) $$S_{\theta ,g}=\frac{1}{n_{analyte}\cos\left(\theta \right)}\left[{\left\{\frac{Re\left(\varepsilon_{metal}\right)}{Re\left(\varepsilon_{metal}\right)+{\left(n_{analyte}\right)}^{2}}\right\}}^{\frac{3}{2}}-\sin\left(\theta \right)\right]$$

(4) $$S_{\lambda ,g}=\frac{\frac{m\lambda }{n_{analyte}\Lambda }+\sqrt{\frac{Re\left(\varepsilon_{metal}\right)}{Re\left(\varepsilon_{metal}\right)+{\left(n_{analyte}\right)}^{2}}}\frac{{\left(n_{analyte}\right)}^{2}}{\left|Re\left(\varepsilon_{metal}\right)+{\left(n_{analyte}\right)}^{2}\right|}}{\frac{m}{\Lambda }+\frac{{\left(n_{analyte}\right)}^{3}}{2\sqrt{Re\left(\varepsilon_{metal}\right)}{\left|Re\left(\varepsilon_{metal}\right)+{\left(n_{analyte}\right)}^{2}\right|}^{\frac{3}{2}}}\frac{dRe\left(\varepsilon_{metal}\right)}{d\lambda }}$$

where *θ* is the incident angle of the light illuminated on the diffraction grating, Λ is the diffraction grating pitch, and *m* is an integer representing the mode number. In a parametric study comparing angular prism-based and diffraction grating-based SPR sensors [49], the sensitivity of SPR sensors using a diffraction grating was not significantly different from that of Kretschmann configuration-based SPR sensors. However, grating-based SPR sensors that perform detection by measuring spectral shifts have a lower sensitivity than SPR sensors that use a prism.

Several preliminary and practical studies of diffraction grating-based SPR sensors in biological and chemical detection have been performed. Bier et al. applied a diffraction grating coupler and SPR for real-time monitoring of nucleic acid hybridization [54]. In feasibility studies using three different DNA targets, the grating coupler-based SPR sensor was shown to be suitable for measuring various biological components, ranging from DNA to a virus. In addition, the development of a miniature SPR sensor based on a diffraction grating has progressed in connection with the investigation of grating structure fabrication techniques and the implementation of microfluidic in vitro analytical devices [55,56]. Piliarik et al. introduced a miniaturized and commercially effective SPR sensor using diffraction grating couplers, as illustrated in Figure 1d [47]. In a preliminary study using solutions with various refractive indices and a practical application for oligonucleotide measurements, the grating-based compact SPR sensor exhibited a sensing performance (3 × 10^−7^ RIU) comparable to that of commercial SPR instruments.

#### 2.2.3. Fiber-Type Method for Miniaturization

An optical fiber is a device that transmits photonic signals with low loss. The fiber is composed of thin glass or plastic. The internal and external refractive indices of the optical fiber can generate TIR, and the light signal goes in a certain direction with a high transfer efficiency, which maintains the TIR. The TIR generation is similar to the prism and diffraction grating methods described in the previous sections. Therefore, an appropriately treated optical fiber can be employed to detect biological and chemical changes via SPR (Figure 1e) [57]. For instance, Cennamo et al. fabricated a fiber-based SPR sensor by removing the cladding on a selected region, while a thin gold film was sputtered above the region [58]. The initial resonance angle of TIR is determined by the characteristics of the optical fiber. Thus, there is difficulty in the application of SPR sensing using angular interrogation. Therefore, fiber-based SPR sensors are generally based on the measurement of spectral changes in resonance conditions.

The fiber-based SPR sensor has several advantages in comparison with prism- and diffraction grating-based SPR sensors. First, the fiber-based SPR sensor can be employed in miniaturized sensors. In addition, the fiber-based SPR sensor can be inserted into and perform direct detection in an extremely small sample. The feasibility to investigate disposable and cost-effective sensing devices is also a merit of the fiber-based SPR sensor. Therefore, some fiber-based SPR sensors have been developed and commercialized. Slavík et al. suggested an SPR sensor consisting of an optical fiber that was bent to come into contact with the sample to measure staphylococcal enterotoxin B with a highly improved detection speed and sensitivity (in the range of ng/mL) [59]. Pollen et al. investigated a fiber-based SPR sensor that was compatible with a commercial syringe needle to detect DNA hybridization processes, as illustrated in Figure 1f [48]. With the integration of computer-controlled sensor positioning stages, the prototype of an automatic multi-sample DNA hybridization instrument was established, providing the feasibility of cost-effective biological activity monitoring systems.

**Figure 2:**
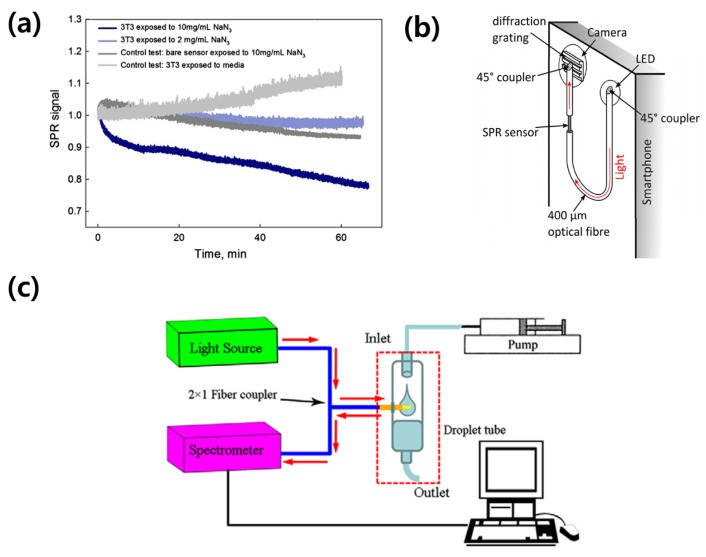
Advanced fiber-based SPR sensors. (**a**) SPR signals when cells cultured on the fiber-optic based SPR sensor (inlet in (a)) for cellular chemical reaction measurements were exposed to sodium azide (NaN_2_) with several concentrations (10, 2 and 0 (control) mg/mL). A SPR signal of 10 mg/mL NaN_2_ solution without cells was also measured for another control data (reproduced from [61] with permission from Elsevier); (**b**) A schematic of the fiber-based SPR sensor implementable to a smartphone. An inlet graph illustrates changes of SPR signals by varying refractive indices on the sample node (reproduced from [64] with permission from the Optical Society); (**c**) a fiber-optic SPR sensor coupled to a droplet tube in a tested glucose infusion instrument. In preliminary studies, the SPR sensor can be employed for monitoring status in various medical infusion instruments (reproduced from [66] with permission from IOP Publishing, a publishing company of the Institute of Physics).

Several research groups have suggested high-sensitivity detection of activities and chemical responses in cells attached to fiber-based SPR sensors [60]. Shevchenko et al. introduced a fiber-based, gold-coated SPR sensor integrated with multi-well cell culture plates [61]. In real-time measurements of SPR signals before and after the exposure of cell culture media to multiple sodium azide (NaN_2_) concentrations, the feasibility of detecting chemical reactions in cultured cells using a fiber-based SPR sensor was well established, as displayed in Figure 2a. Peng et al. investigated a fiber-optic SPR sensing platform including a web camera for acquiring both SPR sensing and imaging data [62,63]. In practical applications of fiber-optic SPR sensors, several research groups have introduced the integration of fiber-optic sensors with a smartphone. Bremer and Roth applied a light-emitting diode (LED) and a camera in a smartphone as an illuminator and a spectroscopic detector in fiber-based SPR sensors, as illustrated in Figure 2b [64]. For the use of a smartphone camera in spectroscopic measurements, a diffraction grating was installed. Liu et al. also introduced a two-channel (sample and reference channels) fiber-based SPR biosensor platform using a smartphone [65]. In clinical use, J. Wu et al. introduced a feasibility study of a glucose infusion monitoring device using a fiber-based SPR sensor, as shown in Figure 2c [66]. In that study, the fiber-optic SPR sensor could detect micro-sized air bubbles in a glucose infusion system supplying glucose to a patient, and the infusion system informed the medical staff of necessary actions when the SPR sensor detected bubbles. In a similar method, S. Qian et al. suggested a peristaltic pump with a fiber-optic SPR sensor that can detect saccharide concentration changes in infusing media [67].

## 3. Advanced Techniques to Improve the Sensing Performance of SPR Sensors

### 3.1. LSPR Sensors

Among several SPR phenomena, LSPR denotes SPR on the surface of nanostructured substrates or nanoparticles with a large concentration in a well-confined area [68,69,70,71,72,73]. The resonance conditions in LSPR based on nanostructures or nanoparticles are more responsive than those of SPR. Therefore, LSPR has been actively applied to develop high-resolution imaging [74,75,76,77,78] and high-sensitivity sensing systems [79,80,81,82] based on the proportional relationship between concentrated fields and the sensitivity [83]. In fact, to find optimum condition of field localization, there have been reported lots of tries with varied nanostructures such as nanoslits or nanoholes. For instance, high sensitive detection based on enhanced optical transmission generated by nanoholes is one of the representative biosensors [84,85]. In this section, performance-improved sensors based on the LSPR phenomenon using periodic/non-periodic nanostructured substrates or nanoparticles are described.

**Figure 3:**
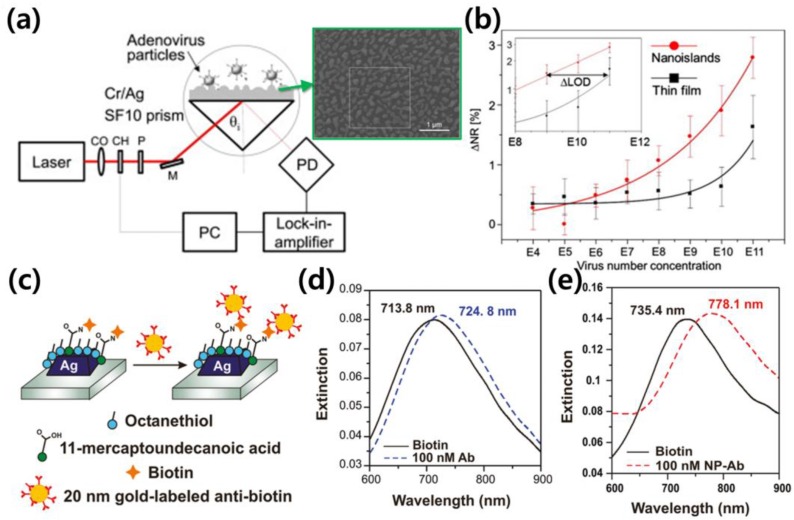
(**a**) The schematic image of the LSPR sensor which is utilizing the non-periodically distributed nanostrucutres is presented. The randomly positioned nanostructures were used for efficiently localized the plasmonic field. The SEM image of the nanostructures is shown in green box (reproduced from [86] with permission from Elsevier); (**b**) The changing of resonance peak was recorded for sensing the refractive index changes when the concentrations of virus get increased on the two kinds of substrate. The material feature was measured more sensitively on the nanoisland-existed substrate than on thin film substrate as presented in graph (reproduced from [86] with permission from Elsevier); (**c**) To amplify the wavelength shift of LSPR sensor, gold nanoparticle-labeled antibodies were used. The conjugation of antibody-nanoparticle improves the observed binding constant and this amplification strategy provides a way to improve the sensitivity of plasmon-based bioassays, paving the way for single molecule-based detection and clinically relevant diagnostics (Reprinted with permission from [92]. Copyright 2011 American Chemical Society); (**d**) The maximum Δλ was 11 nm after binding of native antibiotin (dashed blue) whereas 42.7 nm of Δλ was monitored after binding of antibiotin-labeled nanoparticles (red dashed line) as illustrated in (**e**) (Reprinted with permission from [92]. Copyright 2011 American Chemical Society).

H. Yu et al. enhanced the sensitivity of the sensor using nanoisland structures [86]. The resulting enhancement was exploited in conjunction with an efficient overlap between the target and the near-field distribution produced by the nanoislands. The measured changes in refractive index, which occurred due to an increased virus concentration on the nanoisland substrate, performed better than when measured on a thin film substrate, as illustrated in Figure 3a,b. Song et al. presented a detailed calculation of efficient near-field distributions produced by nanoisland structures based on SEM images and demonstrated an efficiently localized near-field distribution on nanoislands [87]. Cattoni et al. utilized arrays of plasmonic nanocavities for biosensing, which led to a refractive index sensitivity of 405 nm/refractive index unit (RIU) and an FOM of ~21 [88]. Haynes et al. introduced a large variety of nanoparticle structures and well-ordered 2D nanoparticle arrays using nanosphere lithography (NSL), and the sensitivities of each feature were probed using an LSPR sensor [89]. Tanaka et al. utilized high-refractive-index silver nanoparticle sheets on a metal substrate for a high-sensitivity SPR sensor [90]. In that study, the angular shifts increased with the number of Ag nanoparticle sheet layers, improving the angular sensitivity compared with a conventional SPR sensor. Kim et al. utilized self-templating genetically engineered M13 bacteriophage, which is an His-Pro-Gln peptide bound (HPQ) phage, as an optical structure to enhance the sensitivity and selectivity of an SPR sensor [91]. By nematically aligning HPQ phage on a metallic substrate to act as an optical structure, the orientation of the HPQ phage was found to affect the sensitivity in near-field confinement. According to the report, the nematically aligned HPQ phage demonstrated the highest sensitivity at the orientation perpendicular to the incident light source. In addition, the selectivity was improved via a genetically engineering process of M13 bacteriophage to selectively bind with target biomolecules, which enables the detection of analytes at extremely low concentrations, down to the femto-molar level, in real-time.

Hall et al. amplified the wavelength shift of the LSPR sensor using gold nanoparticle-labeled antibodies and demonstrated an up to 400% amplification of the shift [92]. The antibody-nanoparticle conjugation improves the observed binding constant, and this amplification strategy provides a way to improve the sensitivity of plasmon-based bioassays, paving the way for single molecule-based detection and clinically relevant diagnostics. The improved sensitivity was demonstrated, as the maximum Δλ (resonance wavelength shift) values were 11 nm after (dashed blue) the binding of native antibiotin (Figure 3d), whereas a Δλ of 42.7 nm was observed after the binding of antibiotin-labeled nanoparticles, as illustrated in Figure 3e. Kim et al. employed gold nanoparticles and a gold nanoisland chip to enhance the monitoring sensitivity [93]. Target molecule concentrations higher than 79 nM can be detected through biological binding, and these methods show an 11-fold sensitivity compared to detection without gold nanoparticle conjugates. He et al. used Au particle tags and achieved more than a 10-fold increase in angle shifts. The authors described several factors that contribute to sensitivity enhancement by employing gold nanoparticles in that research: (1) the particular binding event increases the mass on the surface compared with a bare target molecule; (2) the refractive index of the gold particle is much higher than that of the biomolecules; (3) the SPR response may increase via electromagnetic coupling between the metallic nanoparticles and the film by influencing the plasmonic mode propagation [94]. Springer et al. researched the response enhancement of an SPR sensor based on the size of the gold nanoparticle [95]. Law et al. achieved a greater than 20-fold sensitivity improvement in a phase-sensitive SPR biosensor by utilizing gold nanorods as powerful amplifying labels [96]. The primary reason for the performance improvement was revealed as nanorod-to-film plasmonic coupling based on numerical simulations. Kwon et al. induced larger spectral shifts by changing the refractive index of the sampling area, enabling the detection of protein biomarkers at attomolar concentrations using antibody-conjugated nanoparticles of various shapes and sizes (approximately 40 to 50 nm in diameter) [97].

### 3.2. LRSPR Sensors

**Figure 4:**
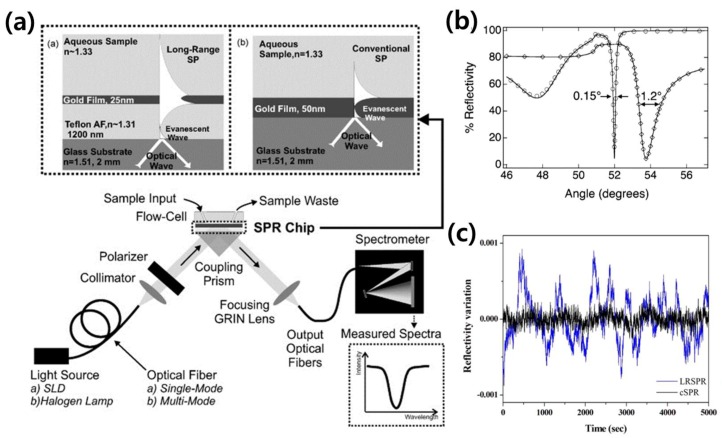
(**a**) The LRSPR sensor exploiting a layer of 1200 nm-thick Teflon AF between glass substrate and gold film is depicted. The deeply penetrated SP propagation compare to conventional SPR sensor is shown (reproduced from [102] with permission from Elsevier); (**b**) The sensing ability of LRSPR which has Cytop layer between SF10 prism and gold film is shown in (**b**). The 1.2° of FWHM (full width at half maximum) at resonance angle deep get extremely sharpen by adding Cytop layer (Reprinted with permission from [100]. Copyright 2005 American Chemical Society); (**c**) The status of adherence of cell was measured in dynamic fluctuation. By adopting the LRSPR sensor method, the cell adherence was observed in a larger fluctuation compare to conventional method (Reprinted with permission from [101]. Copyright 2014 American Chemical Society).

For refractometer applications using an SPR sensor, the refractive index change of an effective thin medium layer at the metallic sensing surface is detected by probing the spectral shifts of the resonance dip via wavelength or angular interrogation. The sensitivity is determined via the resonance dip because the positions of the resonance dip play an important role in defining the sensitivity of the optical sensor as a result of resonance shifts. To improve the sensitivity of the SPR sensor, the width of the resonance feature must be decreased, which reduces the ambiguity of the resonance position. While the previously described method aimed to improve the sensitivity of the sensor by enlarging the resonance shifts due to a change in refractive index, this section describes a method for improving the sensitivity of the sensor by reducing the width of the resonance dip. The LRSPR sensor was suggested by Matsubara et al. in 1990 as a method to enhance sensitivity by adding an additional layer between the prism and the metallic film to the conventional Kretschmann geometry-based SPR sensor, resulting in significantly reduced peak widths [98]. According to the authors, the reduced peak widths were one-third of those obtained using a conventional SPR sensor when the thickness of the additional layer was optimized. The value of the imaginary part K_i_ of the surface plasmon wavenumber is a key factor because it determines the sharpness of the resonance dip. To obtain a sharp resonance dip, a small K_i_ value of the surface plasmon must be excited. K_i_ is dependent on the composition of the sensing metal film. For this reason, many studies based on SPR sensors employ silver films to enhance sensitivity by obtaining a sharp resonance peak, as silver has a small damping coefficient. In addition, the layer structures located around the metallic film at the sensing surface can affect K_i_, which can be used to improve absorption properties [99]. The LRSPR sensor specializes in improving sensitivity by employing additional layers to reduce the resonance dip width. In addition, this sensor possesses a higher electric field strength and a deeper penetration depth [100]. Vala et al. experimentally investigated the performance of an LRSPR sensor compared to a conventional SPR sensor. As depicted in Figure 4a, a 1200-nm-thick Teflon AF with a refractive index very close to that of an aqueous sample was added between the metallic sensing surface and the glass substrate, and the penetration depth of LRSPR was found to be much deeper than that of conventional SPR. The penetration depth for LRSPR is typically 500–1000 nm while that of conventional SPR is approximately 100–200 nm [101]. In that study, the LRSPR sensor sensitivity was confirmed to be 59,000 nm/RIU, corresponding to a medium refractive index change approximately 8 times greater than that of a conventional SPR sensor [102].

Wark et al. added a Cytop layer between an SF10 prism and a gold film for LRSPR. Thus, the fabricated multilayer substrate consisted of the SF10 prism/Cytop/gold/medium layer structure, in that order [100]. As shown in Figure 4b, the width of the resonance curve becomes extremely narrow compared with that of a conventional SPR sensor. The full width at half-maximum of the SPR curve measured for a conventional SPR chip is confirmed to be 1.2°, while the resonance dip measured for the LRSPR chip is more symmetric in shape, with a full width at half-maximum of 0.15°.

Yang et al. adopted an LRSPR sensor to non-invasively measure dynamic fluctuations in adherent cells [101]. The substantially larger probing depth toward the medium and sensitivity compared with that of conventional SPR helped to sensitively measure the micro-motion of cells. Significant optical fluctuations can be observed using LRSPR based on the variation of reflectivity, whereas very few fluctuations were obtained using a conventional SPR sensor, as illustrated in Figure 4c.

### 3.3. Double-Metal-Layer LSPR Sensors for In Vitro Cell-Based Biosensors

#### 3.3.1. Applications of SPR and LSPR for In Vitro Cell Based Biosensor

In the previous sections, we have described various SPR sensors and several sensitivity-enhancing techniques based on advanced nanotechnology for biological, biomedical, chemical, and molecular measurements. In this section, we explore applications of SPR for in vitro cell-based biosensors. Various in vitro cell-based assays have been actively studied for understanding disease processes [103,104], developing drugs to diseases [105,106], and analyzing metabolic activities [107]. Along with an increasing number of in vitro cell-based studies, related experimental devices and instruments have also been widely developed, such as microfluidic biochips [108,109,110], automated cell culture systems [111], and analytical platforms to measure specific cellular activities [112,113,114]. For this approach and with the merit of label-free measurement, several research groups have investigated analytical approaches based on SPR-based sensing and imaging techniques for cell-based assays. Peterson et al. developed an SPR imaging instrument for label-free, real-time imaging of cells and their extracellular matrix [115]. The processes of cell growth and extracellular matrix formation between neighboring cells were measured using an SPR imaging system. This research group suggested high-resolution SPR imaging of a single cell using an objective lens with a high numerical aperture (NA) and a light modulator to generate light [116]. Robelek and Wegener demonstrated that an SPR sensing technique can be applied for temporal measurements of cell volume changes, which is a key parameter in cell pathology [117]. Sefat et al. used an SPR imaging microscope to analyze cell surface attachments and interactions between a micro-patterned extracellular matrix and cells cultured on the matrix [118]. Quantitative comparisons of cell-protein interactions under different extracellular matrix conditions with various pattern sizes and protein types were achieved using an SPR imaging microscope. Wang and co-researchers introduced applications of SPR imaging for label-free monitoring of membrane protein binding processes in a single cell [119] and high-sensitivity measurements of electrical impedance distributions and changes in cultured cells using induced modulated AC potentials [120].

In SPR detection and imaging in cell-based assays, there are two important issues: (1) enhancing performance (i.e., sensitivity or resolution) for a specific cellular activity and (2) procuring biocompatibility in cells cultured on the surface in SPR configurations. To improve sensitivity in cell-based assays using SPR, LSPR, which is based on nanostructures or nanoparticles as described in the previous sections, has been applied. For instance, Kim and Kim applied plasmonic modulations by introducing nanoscale grating structures to enhance the resolution in SPR imaging [121].

#### 3.3.2. Double-Metal-Layer SPR Sensor for In Vitro Cell Based Biosensor

The issue of biocompatibility in cells is most closely related to the sensor surface material. In general, silver (Ag) and gold (Au) have been primarily used as materials for SPR sensors. Ag-based SPR sensors are well known to present high sensitivity because of their narrow resonance dip. Although it presents a high sensitivity, silver has limitations because it is vulnerable to oxidation and is chemically unstable. Because silver-based chips can be easily oxidized by the surrounding environment, such as by water, they are difficult to apply for long-term detection. With respect to Au-based SPR sensors, gold is chemically stable, but has a lower sensitivity towards analytes than Ag-based sensors. Thus, appropriate materials should be exploited in SPR sensors for the precise sensing application. Hence, there are many efforts to enhance the specific functions, which are can be obtained from choosing a metallic material, of SPR sensor using double-metal-layer structure.

**Figure 5:**
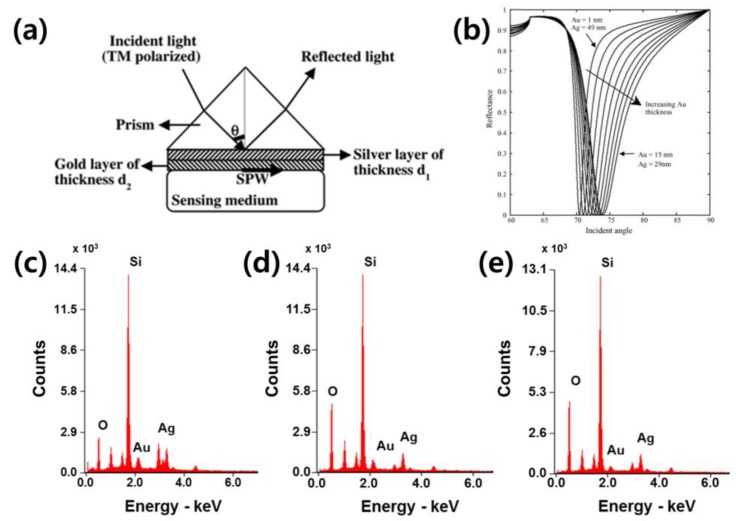
(**a**) A schematic image of Double-metal-layer LSPR sensors is animated. The two-different thicknesses of double-metal-layer metallic layers are presented between prism and sensing medium (reproduced from [122] with permission from Elsevier); (**b**) The thicker gold–thinner silver film coated substrate shows a broader FWHM of resonance dip, thereby it lower sensitivity (reproduced from [123] with permission from Elsevier). It would be better to choose thicker silver film with thin gold film for enhancing the sensitivity. The EDS spectra of the double-metal-complex substrates are shown when the deposition rates are (**c**) 1:1, (**d**) 1:2 and (**e**) 1:3.

Sharma and Gupta demonstrated a theoretical analysis of sensitivity for a sensor based on an Ag-Au bimetallic layer substrate. The numerical calculation is based on the SPR theory and the Drude model of metals. The bimetallic layer chip is one development that simultaneously takes advantage of two materials, benefiting from the biological/chemical stability of gold and the high sensitivity of silver [122] (Figure 5a). Ong et al. explored the optimum configurations of silver and gold films as an integrated bimetal layer for enhancing detection sensitivity while maintaining a higher sensitivity due to the refractive index change of silver and the high chemical resistance of gold [123]. As shown in Figure 5b, a thicker gold-thinner silver coating presents a broader FWHM of the resonance dip; thus, it has a lower sensitivity. In this research, the bimetallic configuration of a 42-nm-thick silver film and a 5-nm-thick gold film is sufficient to protect the silver from being chemically unstable.

#### 3.3.3. LSPR Sensor Using Double-Metal Complex for In Vitro Cell Based Biosensor

For now, we suggest another LSPR method using a double-metal-complex substrate instead of previously introduced layer by layer structure. This method also can obtain both high biological/chemical stability and sensitivity simultaneously with more time saving and simpler fabrication sequence. Furthermore, it is notable that this method enables the fabrication of nanostructures on that double-metal-complex.

To fabricate the double-metal-complex film on the glass substrate, two metallic materials were simultaneously deposited in 60-nm-thick on the SF-10 glass at different deposition rates (gold:silver is 1:1, 1:2 and 1:3) with a 5-nm-thick Ti adhesion layer using an evaporator. After that, the nanowire structures are fabricated on a metallic film to obtain the LSPR effect by localizing the electromagnetic field on the structures. The nanowire patterns were defined via electron-beam (e-beam) lithography. For the e-beam lithography, we used a positive e-beam resist, poly (methyl methacrylate) (PMMA) (AR-P 679.04, Allresist GmbH, Strausberg, Germany). The nanowire was patterned with a 250-nm width and a 1-μm period. The resist was developed, followed by the deposition of a 40-nm-thick double-metal-complex, and then, the remaining e-beam resist was removed by a lift-off process. Finally, the gold-silver nanostructure substrate was fabricated. The double-metal-complex based film type substrate fabricated at different deposition rates were examined using energy dispersive spectroscopy (EDS) for metal composition analysis. The nanowire patterned double-metal-complex based substrate was characterized using SEM. The corresponding EDS results confirmed the presence of Au in the double-metal-complex (Table 1). The composition of substrates are analyzed by EDS analysis as 55.9 Au-44.1 Ag at.%, 50 Au-50 Ag at.%, 23.8 Au-76.2 Ag at.% when fabricated at different deposition rate of gold and silver, such as 1:1, 1:2 and 1:3 Au and Ag, respectively (Figure 5c–e).

**Figure 6:**
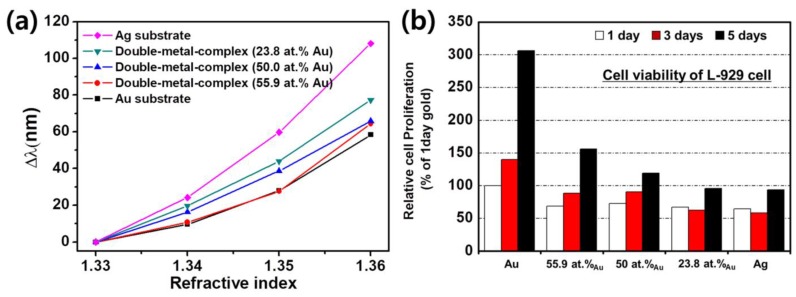
(**a**) The measured resonance peak shifts from RI differences on nanowire-patterned double-metal-complex substrates with various compositions. As the resonance peak shifts show, the patterned silver substrate presents the highest sensitivity. For the patterned double-metal-complex substrates, the sensitivity was improved as the composition of gold is decreased; (**b**) The cell viability assay results for testing the cell affinity of each substrate when culturing the cell on those substrates for a day, 3 and 5 days. The higher proportion of gold substrate enhances cell viability, also, the cell viability of the 23.8% gold substrate was similar to that of the silver substrate.

To test the performance of the sensor, we used target samples with diverse refractive indices, prepared from a % *v*/*v* mixture of water and ethanol (*n* = 1.33, 1.34, 1.35, 1.36). Figure 6a shows the measured spectral changes from 0.01 refractive index (RI) difference on nanowire-patterned double-metal-complex substrates with various compositions. The sensitivities of the gold and silver nanowire-patterned substrates are observed as 1930 nm/RIU and 3600 nm/RIU, respectively. As the amount of resonance peak shifts shows, the patterned silver substrate presents the highest sensitivity. For the patterned double-metal-complex composed of gold and silver substrates, the sensitivity was improved as the composition of gold is decreased; for instance, values of 2580, 2200 and 2100 nm/RIU were observed for the substrates with 23.8, 50 and 55.9 at.% Au.

To examine the cell affinity of the fabricated double-metal-complex substrates, we measured the survived cell culturing on those substrates. For the assay, L929 cells were used and were incubated for a day, 3 and 5 days culturing on those substrates. For the accuracy of the experiment, the exactly same number of cells, 104 cells/mL, were adjusted on the double-metal-complex substrates. Then, the plate was incubated at 37 °C in a 5% CO_2_ atmosphere for one, three and five days. After that, cell was fixed by acetaldehyde, and the survived number of cell was counted using a microscope to determine the cell viability of the substrates (Figure 6b). The data were compiled based on the standard setting, which is defined by the number of live cells cultured on the gold substrate for 1 day as 100%. According to our results, the higher composition of gold substrate serves the higher cell viability. On the 5th day, we measured the number of cells cultured on substrates with gold composition ratios of 100, 55.9, 50, 23.8 and 0 at.% Au, and the cell viability was revealed to be 300%, 156%, 120%, 95% and 93%. It means that the higher proportion of gold substrate enhances cell viability, also, the cell viability of the 23.8% gold substrate was similar to that of the silver substrate.

For the gold-silver double-metal complex substrate, we confirmed that an enhanced plasmonic effect and a higher cell viability can be achieved at the same time. Using the double-metal complex substrate with nanowire structures, we can obtain significantly improved resonance shifts, maximally 2580-nm resonance wavelength shifts for a refractive index change of 1, using a conventional SPR sensor system while maintaining high cell viability. These results demonstrate that the sensitivity of the SPR sensor system can be dramatically enhanced by depositing the gold and silver materials at appropriate rates. In addition, the cell viability can be enhanced compared with a silver-only substrate while still sustaining high sensitivity. When comparing the SPR sensitivity measured for the gold and gold-silver 55.8 at.% Au mixture, an approximately 1.1-fold enhancement was observed as the maximum case. In addition, the resonance shifts measured on the gold-silver 55.8 at.% Au mixture substrate shows more distinctive spectra compared with that measured on the silver substrate. Consequently, a substrate with two deposited metallic materials, i.e., gold-silver 55.8 at.% Au, is more useful than a gold or silver substrate.

## 4. Concluding Remarks

In this paper, we explore the fundamentals, configurations, and practical realizations of SPR sensors. In addition, several advanced SPR sensors based on nanotechnology were introduced: (1) LSPR sensors using nanostructures or nanoparticles; (2) LRSPR; and (3) double-metal-layer SPR sensors for additional performance improvements. In addition, we suggest a practical method for achieving high sensitivity in LSPR sensors while also maintaining high biocompatibility. Importantly, the improvement of SPR sensors has progressed with several aims: (1) enhancing the sensitivity; (2) securing selectivity to specific targets; and (3) increasing biocompatible stability. Furthermore, additional advancements in SPR sensors to secure an efficient performance should continue and several research groups proposed SPR sensors with highly improved performances using advanced fabrication and converged techniques, for instance, symmetric insulator-metal-insulator substrate based LRSPR [124], sensing performance improvement using a top dielectric nanoscale layer [124,125], a spectropolarimetric SPR sensor [126,127], and combined exciting localized and extended surface plasmon sensors with ultrahigh sensitivity [128,129]. On the other direction, the miniaturization of SPR sensors is also needed to increase utilization, and research groups have reported several prototypes of miniaturized SPR sensors [130,131,132,133,134,135,136]. For instance, Shin et al. developed and commercialized a miniaturized SPR sensing instrument based on light illumination using a rotating mirror (MiCo SPR Nano, MicoBioMed Co., Ltd., Anseong, Republic of Korea) [137]. In addition, SPR sensors coupled with information communication technology should be needed to follow the trend of interconnections between devices in the 4th industrial revolution. A few research groups have performed feasibility studies to integrate SPR and enhanced plasmonic sensors in a smartphone for acquisition, data storage, analysis, and data transfer to external devices or databases [138,139,140]. As technologies advance, such as low-powered electronic components and high-speed wireless transfer techniques, these developments in SPR sensors are expected to accelerate further.

## Figures and Tables

**Table 1:** The numerical EDS results of the double-metal complex substrates.

Ratio of Deposition Rate (Gold:Silver)	wt %		at.%	
	Au	Ag	Au	Ag
gold	100	0	100	0
1:1	69.9	30.1	55.9	44.1
1:2	64.6	35.4	50.0	50.0
1:3	36.3	63.7	23.8	76.2
Silver	0	100	0	100
